# Selected Updates in Anti-arrhythmic Drug Therapy and Anticoagulants: 2024

**DOI:** 10.19102/icrm.2025.16018

**Published:** 2025-01-15

**Authors:** James A. Reiffel

**Affiliations:** 1 Columbia University, New York, NY, USA

**Keywords:** Anti-arrhythmic, antisense oligonucleotide, factor XI, factor XIa, monoclonal antibody

As the Pharmacologic Insights Section Editor of the *Journal of Innovations in Cardiac Rhythm Management*, I have the opportunity each year to write a commentary discussing selected papers or pharmacologic therapy highlights from the recent past for this section if such seems appropriate. For 2024, I have elected to comment on the following: (1) observations regarding anti-arrhythmic drugs (AADs) that were reported in 2023–2024, most relating to safety, and (2) the possible new horizon in oral anticoagulation via factor XI or XIa inhibitors. I picked these areas in case they have not yet come to the attention of the journal’s readership.

## Selected 2023–2024 observations regarding anti-arrhythmic drugs

### Papers regarding overviews of anti-arrhythmic drug therapies

**Figure 1:**
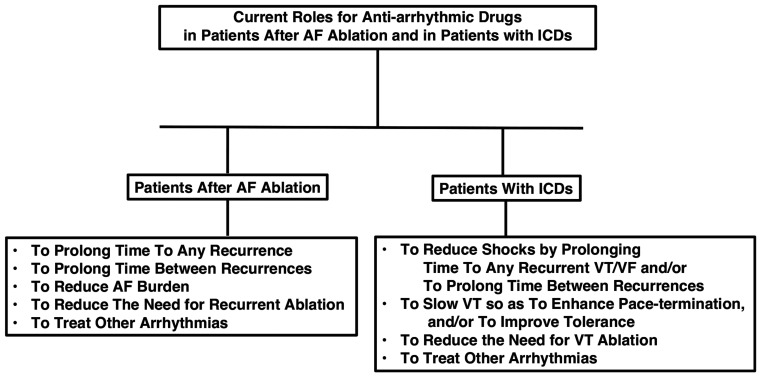
Roles for anti-arrhythmic drugs in patients after catheter ablation for atrial fibrillation or in patients with implantable cardioverter-defibrillators. *Abbreviations:* AF, atrial fibrillation; ICD, implantable cardioverter-defibrillator; VF, ventricular fibrillation; VT, ventricular tachycardia.

Two specific papers discuss the recent advances in AAD therapy and where AAD therapy might move forward from the present.^1,2^ Both concisely but insightfully comment on the path of prior AAD development as well as what the future might hold for their further development. Kowey and Naccarelli^1^ astutely pointed out that newer treatment technologies, such as ablation, have led to the need to address residual or resultant arrhythmias that can follow such procedures. In fact, as I noted earlier this year in an editorial in *Circulation*,^3^ AAD therapy has been used in post-ablation trials in up to 60% of patients despite the absence of this fact being noted in the abstracts and conclusions of the trials’ publications. Moreover, the fact that ongoing AADs post-ablation can markedly reduce recurrences of atrial fibrillation (AF) and the need for repeat ablation was dramatically shown in the PulmOnary vein isolation With vs. without continued antiarrhythmic Drug trEatment in subjects with Recurrent Atrial Fibrillation (POWDER AF) trial in 2018.^4^ AADs for this purpose have not yet been systematically studied and compared, but such trials would be greatly welcomed. Kowey and Naccarelli also noted the ongoing need for AADs in implantable cardioverter-defibrillator patients with frequent events, as our current choices for this population remain limited. Trials with an investigational agent, azimilide, were encouraging in this arena, but the drug never came to the market for logistical and other reasons. Nonetheless, in the words of Kowey and Naccarelli, “AAD treatment has maintained an important role in clinical practice” **(Figure 1)**.

While I believe this will continue to be true moving forward, it will likely take the form of new agents and new methods of administration. Regarding the latter, Saljic et al.^2^ pointed out that new methods of administration may take the form of intranasal or inhaled delivery. This is being studied for flecainide when used for the acute termination of new-/recent-onset AF and for the investigational drug, etripamil, a verapamil congener when used for the acute termination of paroxysmal supraventricular tachycardia.^2^ Early studies with these products/delivery systems have been encouraging but are not yet ready for U.S. Food and Drug Administration approval.

New AADs under investigation include those that focus primarily on channels present in the atria. The now greater than a decade of experience with vernakalant in Europe has revealed the value of this approach. One such new “atrial-selective” agent with results reported in 2024 is a small conductance Ca^2+^-activated K^+^ channel blocker, the AP30663 product of Acesion Pharma (Zealand, Denmark). This compound appeared successful in its phase 2 clinical trial,^5^ where the drug appeared favorable for the conversion of AF as compared to placebo. The primary endpoint was conversion to sinus rhythm within 90 min. This was achieved in 42% with a dose of 3 mg/kg and 55% with a dose of 5 mg/kg versus 0% on placebo. Adverse event (AE) rates were the same for the drug and placebo, despite a mild, transient increase in the QT interval on the active drug. Further studies with subsequent-generation agents are underway by Acesion Pharma.

Also of potential value going forward are AAD combinations. Although the 2024 European Society of Cardiology (ESC) Guidelines for the Management of Atrial Fibrillation^6^ state that “combinations of AADs are not recommended,” the HARMONY trial^7^ previously demonstrated that combinations can be both beneficial and better than either agent alone. In the HARMONY trial, the combination of dronedarone plus ranolazine successfully reduced AF burden as determined by implanted devices—and did so to a greater extent than either agent at the same doses did alone. Additional information regarding the clinical utility (rationale, indications, and experience) of these results and other AAD combinations can be found in the 2023 review of this subject by Reiffel et al.^8^

Finally, in the overview category, I feel it appropriate to note the paper by Spears et al.^9^ from 2023 regarding a global survey of cardiologists that demonstrated that physicians continue to make practice choices that deviate from those recommended in the guidelines promoted by our major cardiac organizations. This survey was from the AIM-AF group.^9^ While there can be many reasons for a particular physician facing a particular patient to make a patient-specific rather than guideline-suggested therapeutic choice, we should continue to recognize that often the deviance is from inadequate awareness of or familiarity with the guidelines (which change over time) and that further continued medical education is an ever-ongoing need. More details about the discordance between practice and guideline recommendations can be found in two older papers^10,11^ as well as in the aforementioned report by Spears et al.^9^

## More specifically focused reports of anti-arrhythmic drug therapy from 2023 and 2024

### Class IC anti-arrhythmic drugs

One of the areas of deviation from guidelines noted in the paper by Spears et al.^9^ was the use of class IC AADs in patients with heart failure (HF), including some with HF with reduced ejection fraction (HFrEF). In 2023–2024, several additional publications dealt with this specific issue. As has been the case with virtually all guidelines in the aftermath of the Cardiac Arrhythmia Suppression Trial (CAST),^12^ even the extremely recent ESC’s 2024 AF guidelines^6^ recommend against the use of class IC AADs in the setting of coronary artery disease (CAD) or HF. The 2024 ESC guidelines directly state that “flecainide or propafenone is recommended in patients with AF requiring long-term rhythm control to prevent recurrence and progression of AF, excluding those with impaired left ventricular systolic function, severe left ventricular hypertrophy, or coronary artery disease.” Nonetheless, in 2023, based on a retrospective review of data from a 16-year period including over 3400 patients, Kiani et al. reported on the feasibility and safety of flecainide in patients with CAD.^13^ Patients with a history of non-revascularized myocardial infarction (MI) were excluded. Patients were divided into those with non-obstructive versus obstructive CAD, and flecainide-treated patients in each group were compared to a control group treated with sotalol or dofetilide. The primary endpoints were total mortality, ventricular tachycardia, ventricular fibrillation, and/or decompensated HF. Compared to sotalol, the use of a class IC agent led to poorer event-free survival in patients with obstructive CAD but better event-free survival in patients with less obstructive CAD and no adverse outcomes with the IC agent in HF independent of the above. Thus, the authors suggested that class IC agents have a reasonable safety profile in patients with non-obstructive CAD. However, there were notable differences in the groups as might be expected in a retrospective study, including younger age; fewer men; lower burden of CAD; higher left ventricular ejection fraction (LVEF); less renal, pulmonary, and hepatic disease; and differences in concomitant medications in flecainide-treated patients. These confounding factors should limit the extrapolation of these results to other populations. As Naccarelli similarly pointed out in an accompanying editorial,^14^ there were several relevant limitations to this study, thus temporizing its conclusions.

Subsequently, another assessment was published in 2024 by Rillig et al.^15^ This one used a post hoc analysis of the Early Treatment of Atrial Fibrillation for Stroke Prevention Trial (EAST-AFNET 4)^16^ with a focus on primary safety outcomes in the subgroup of 1395 patients treated with flecainide or propafenone in the early rhythm-control group. Twenty-six percent of the class IC AAD patients had stable HF with preserved ejection fraction and 6% had severe CAD, though many were revascularized. Compared to patients treated with another class of AADs, there were no important changes in LVEF or worsened New York Heart Association functional class, and there were no other excess safety issues in those given a class IC AAD. This led the authors to conclude that class IC AADs were reliable and safe in the patients selected for such treatment by the investigators. Obviously, again, the results should be interpreted cautiously knowing the patients were studied retrospectively such that investigators likely chose them carefully in contrast to an analysis from a prospective randomized approach or what might occur in “real-world” practices outside of a clinical study. Accordingly, despite the guidelines stating otherwise, the literature does provide some support for the safe use of class IC AADs in selected patients with CAD, particularly if non-obstructive and/or revascularized without prior MI, but this information must be taken with due consideration of the major limitations of these two studies.

### Class III anti-arrhythmic drugs

Several reports of note regarding class III AADs were also published in 2023–2024. One was a retrospective propensity-matched comparison of dofetilide versus no AAD use in patients with AF plus left ventricular hypertrophy (LVH) (defined as ≥1.4 cm).^17^ In 718 patients, the authors reported that dofetilide was not associated with any increased mortality at 3 years and suggest that their study demonstrated “the safety of dofetilide in this population despite guideline recommendations against its use.” However, before readers of *The Journal of Innovations in Cardiac Rhythm Management* jump favorably toward this conclusion, we should note that, in this trial, the mean LVH was only 1.5 ± 0.2 cm (ie, not severe LVH) and the mean LVEF was 51%–52%. Also, while the authors reported no torsades de pointes, the paper did not discuss any electrocardiogram protocol use during follow-up as part of their analysis. Moreover, the assessment of factors such as medications or lack thereof that could relate to risk with dofetilide was not detailed, no dosing information was provided, and the mean body mass index was around 30 kg/m^2^, which could raise an issue of drug distribution. And, interestingly, there were more AF-related hospitalizations in the dofetilide group than in the placebo group (though all-cause hospitalizations were the same). Thus, while the study’s results are enticing, I believe the authors’ conclusions should have been noted as hypothesis-generating rather than the definitive statement they made.

Further reports involving class III AADs published from 2023 to 2024 include several regarding the use of intravenous (IV) sotalol to facilitate its loading. Liu et al.^18^ reported on a total of 29 patients with “refractory” atrial or ventricular arrhythmias who were loaded with IV sotalol and 20 patients who underwent oral loading. The load was successfully completed in 75.9% of the IV group and 100% of the oral group (although 35% of the oral group required a dose reduction). Ten patients had their IV loading interrupted—seven for bradycardia and three for excess QT prolongation. No patient developed an increased premature ventricular complex burden or a sustained ventricular tachyarrhythmia on either regimen prior to discharge. The IV regimen shortened the length of stay by 2.6 days (1 vs. 3.6 days). Of the 22 patients who completed the IV loading, 10 were discharged on 80 mg twice a day (bid) and 12 were discharged on 120 mg bid. Of the 20 patients who received the oral load, 7 were discharged on 80 mg bid and 13 were discharged on 120 mg bid. The authors concluded that IV sotalol loading has a safety profile similar to oral sotalol loading and significantly shortened the hospital stay—potentially leading to important cost savings. Ideally, however, given the known frequency of torsades de pointes with sotalol, which is in the single-digit percentage points if other risk factors are absent, a much larger trial than 29 patients would be necessary to verify the safety profile reported in this study. Thus, importantly, a second study regarding the expedited loading of IV sotalol, the Prospective Evaluation Analysis and Kinetics of IV Sotalol (PEAKS) registry, was reported in 2024.^19^ In this multicenter study, 167 consecutively enrolled patients were studied (99% for drug initiation, 1% for dose escalation). The sotalol infusion was stopped in two patients for bradycardia or hypotension. Additionally, prior to discharge on target oral doses of 80 mg bid or 120 mg bid (each in about half of the patients), sotalol was stopped for excessive QT interval prolongation in three patients, for bradycardia in one, and for recurrent arrhythmia in two, respectively. The mean length of stay was 1.1 days, with 95% of patients being discharged within 1 night. These results appear to support the prior trial reported earlier; yet, even these authors suggested that more data are still needed regarding the minimal duration required for in-hospital monitoring. Of interest, neither of these trials (or the other previous ones I have seen) compared the IV loading regimen to the accelerated oral loading regimen described by Barbey et al. in 1999.^20^ The latter regimen, which uses a shortened load of oral sotalol guided by rational pharmacokinetic principles, reduced the hospital stay by 1 day in their study. This is a regimen I have used for many years with success. It involves 80 mg every 6 h for four doses followed by 120 mg every 12 h. If excess QT prolongation or untoward bradycardia occurs, the dose is decreased; if not, then discharge is feasible by the third morning.

Finally, with respect to new information regarding sotalol in 2023–2024, a paper by Singh et al.^21^ compared dronedarone versus sotalol in patients with AF using a systematic literature search and network meta-analysis. In this report, dronedarone was associated with a statistically significantly lower risk of all-cause death (hazard ratio [HR], 0.38) than sotalol but no difference in AF recurrence or cardiovascular (CV) death. As in all such analyses, residual biases, such as selection bias, cannot be excluded. Nonetheless, this is similar to a retrospective non-direct comparison of the results with dronedarone in ATHENA^22^ to subgroup analyses of other AADs in the Atrial Fibrillation Follow-up Investigation of Rhythm Management (AFFIRM) trial. In ATHENA, dronedarone reduced CV hospitalization or death, whereas the opposite was seen with class IC AADs, sotalol, and amiodarone compared to no AAD (the rate-control arm) in AFFIRM.^23^ More specifically, in AFFIRM, 729 amiodarone patients, 606 sotalol patients, and 268 class IC patients were matched to rate-control patients. The composite outcome of mortality or CV hospitalizations showed better outcomes in no-AAD patients compared to amiodarone (HR, 1.18; 95% confidence interval [CI], 1.03–1.36; *P* = .02), sotalol (HR, 1.32; CI, 1.13–1.54; *P* < .001), and class IC (HR, 1.22; CI, 0.97–1.56; *P* = .10). There was a non-significant increase in mortality with amiodarone (HR, 1.20; CI, 0.94–1.53; *P* = .15), with the risk of non-CV death being significantly higher with amiodarone versus rate-control patients (HR, 1.11; CI, 1.01–1.24; *P* = .04). Still further comparative information regarding dronedarone versus other AADs is also forthcoming from the same time period. In a data analysis based upon the National Health Insurance Database in Taiwan, Wu et al.^24^ compared dronedarone versus non-dronedarone AADs—specifically amiodarone and propafenone. In the comparison versus non-dronedarone AADs and versus amiodarone specifically, dronedarone was associated with lower all-cause mortality and a lower rate of the major adverse cardiovascular event endpoint (acute MI, ischemic stroke, and CV death). This, too, resembles the indirect comparison noted earlier of the ATHENA versus AFFIRM data. In the dronedarone versus propafenone comparison, there were no differences in primary or secondary endpoints. Once again, being a retrospective comparison, many potentially result-affecting biases could be at play. Nonetheless, the consistency of the results of these several comparisons should be noted regarding the relative safety of dronedarone when choosing a first-line AAD for AF. Recall, however, that dronedarone is contraindicated in severe or unstable HFrEF.

### The possible new horizon in oral anticoagulation via factor XI or XIa inhibitors

We are now in our second decade of experience with the newest generation of oral anticoagulants—the direct thrombin inhibitor, dabigatran, and the factor Xa inhibitors, apixaban, edoxaban, and rivaroxaban. These are commonly termed newer oral anticoagulants or direct oral anticoagulants (DOACs). These agents have come to replace the vitamin K antagonists for most anticoagulation-requiring patients with venous thromboembolism and/or AF. This change is a consequence of the many difficulties of using vitamin K antagonists versus the more uniform dosing, fewer drug or diet interactions, and reduced need for monitoring but a similar or lower bleeding risk and similar or better efficacy associated with the newer agents. Nonetheless, bleeding is still a risk in patients on DOACs, and there continues to be pursuit of additional alternatives with similar if not better efficacy but an even lower bleeding risk. In 2022–2024, studies with such a class of agents, factor XI or XIa antagonists, have come to the fore.^25–28^ Factor XI is required for pathological thrombosis but has minimal impact for physiological hemostasis. It has a primary role in thrombus amplification but only a subsidiary role in hemostasis. Hereditary conditions associated with low or absent factor XI appear to have a lower risk for venous thromboembolism than is present with normal levels. Yet, they also appear not to have an elevated risk for spontaneous bleeding. In 2023–2024, several publications dealing with factor XI or XIa inhibitors appeared, on a background of more preliminary studies during the preceding couple of years. The factor XI or XIa inhibitors include parenterally administered monoclonal antibodies against factor XIa (abelacimab and osocimab), parenterally administered antisense oligonucleotides (such as fesomersen), and orally administered small synthetic molecules (asundexian and milvexian). To date, some of these have been tested in patients with AF in phase 2 trials and are being tested in phase 3 trials (see below).

### Asundexian

In September 2024, the results of the phase 3 Asundexian vs. Apixaban in Patients with Atrial Fibrillation (OCEANIC-AF) trial with asundexian, a direct and selective factor XIa inhibitor, were presented at the ESC Congress in London and simultaneously published ahead of print in the *New England Journal of Medicine*.^29^ OCEANIC-AF was performed after early phase 2 dose-ranging studies with asundexian^30^ as well as a 753-patient two-dose (20 mg and 50 mg once a day) later phase 2 AF trial (the PACIFIC-AF trial).^31^ Each appeared to demonstrate the predicted lower bleeding rates. PACIFIC-AF, which was underpowered regarding the assessment of efficacy, studied asundexian versus apixaban in a multicenter, randomized, double-blind, double-dummy phase 2 trial. It demonstrated that asundexian resulted in lower rates of bleeding as compared to standard-dosing apixaban in the included AF patients. Following these, asundexian was tested against apixaban to assess whether asundexian was non-inferior with respect to stroke or systemic embolism yet superior with respect to major bleeding in history- and demographically defined anticoagulant-requiring patients with AF. The trial was multinational, randomized, double dummy, and double blind. Asundexian was given as 50 mg once daily versus guideline-directed apixaban dosing, and each arm also received matching placebo to allow the double-blind, double-dummy format. The trial enrolled 14,810 patients with a mean age of 74 years and a mean CHA_2_DS_2_-VASc score of 4.3 points, followed for a mean of 155 days. All patients had to have electrocardiogram evidence of AF within the prior year. Unfortunately, because of a greater incidence of thromboembolic events in the asundexian group (1.3% vs. 0.4%; HR, 3.79) that became apparent early, the trial was terminated prematurely, although major bleeding was lower on asundexian (0.2% vs. 0.7%; HR, 0.32). Mortality rates were the same for each agent. However, an ongoing phase 3 trial in non-cardioembolic ischemic stroke patients (OCEANIC-Stroke) is continuing, with results anticipated in 2026.

### Milvexian

Milvexian is another factor XIa inhibitor. As with asundexian, milvexian had initial efficacy and safety assessed in phase 2 studies. The Antithrombotic Treatment with Factor XIa Inhibition to Optimize Management of Acute Thromboembolic Events in Total Knee Replacement (AXIOMATIC-TKR)^32^ and Safety and Efficacy of Factor XIa Inhibition with Milvexian for Secondary Stroke Prevention (AXIOMATIC-SSP) trials,^33^ respectively, studied this agent in patients with thromboembolic disease, where milvexian was compared to apixaban after total knee replacement, and in patients with non-cardioembolic ischemic stroke/transient ischemic attack where milvexian was compared to placebo on background antiplatelet agents. In AXIOMATIC-TKR, dose-ranging milvexian was compared to open-label enoxaparin. Milvexian doses of 100 mg bid or higher led to significant reductions in venous thromboembolism versus enoxaparin with no difference in major or clinically relevant non-major bleeding. In AXIOMATIC-SSP, although the study did not meet the primary efficacy endpoint of reduction in symptomatic ischemic stroke and covert brain infarction on magnetic resonance imaging, there was no dose response noted for major bleeding, and AEs and serious AEs were similar to placebo.

With these as background, milvexian is now being studied against apixaban for stroke prevention in AF in the LIBREXIA trial.^34^ This trial is currently ongoing. It is designed as a multicenter, randomized, double-blind, parallel-group, event-driven, phase 3 trial that will compare milvexian 100 mg bid versus guideline-directed apixaban dosing in 15,500 patients. The primary efficacy endpoint is non-inferiority for the prevention of stroke and systemic embolism. The primary safety endpoint is to evaluate if milvexian is superior to apixaban in reducing major and clinically relevant non-major bleeding. The anticipated trial length is 4 years. Thus, the assessment of factor XI or XIa inhibition remains in its infancy, but stay tuned as its potential seems hopeful. In parallel, milvexian is also being tested in LIBREXIA-Stroke, a phase 3 trial comparing milvexian to placebo in patients with non-cardioembolic stroke. This trial also aims to enroll around 15,000 patients worldwide.

### Monoclonal antibody and antisense oligonucleotides

To date, these compounds have been tested in phase 1 and phase 2 studies in the venous thromboembolism sphere, such as in the FactOr XIa inhibiTion for the pRevention of venOus Thromboembolism in Patients Undergoing Total Knee Arthroplasty (FOXTROT) trial.^35^ In FOXTROT, when osocimab was given postoperatively after knee arthroplasty, it was shown to be non-inferior to enoxaparin; and, when started preoperatively, a 1.8-mg/kg dose showed superiority to enoxaparin. With respect to AF trials with an antisense oligonucleotide agent, the only ones I am aware of so far are A Multicenter, RandomiZed, Active-ControLled Study to Evaluate the Safety and Tolerability of Two Blinded Doses of Abelacimab Compared with Open-Label Rivaroxaban in Patients with Atrial Fibrillation (AZAELA-TIMI 71)^36^ and the Study to EvaLuate the EffIcacy and Safety of AbeLacimab in High-risk Patients with Atrial Fibrillation Who Have Been Deemed Unsuitable for Oral AntiCoagulation (LILAC-TIMI 76).^37^ AZAELA-TIMI 71 evaluated abelacimab versus rivaroxaban in patients with AF and high stroke risk scores. Both doses of abelacimab (90 mg and 150 mg subcutaneously monthly) were superior to rivaroxaban with respect to lower bleeding. The major and clinically relevant non-major bleeding rates were 2.7%, 1.9%, and 8.1%, respectively (*P* < .001 for both doses). There were no differences in the rates of stroke and systemic embolism (1.1%, 1.4%, and 1.0%, respectively). LILAC-TIMI 76 is an ongoing double-blind study comparing abelacimab to placebo in patients who have both AF and a contraindication to standard anticoagulation (NCT05712200) whose primary endpoint is ischemic stroke and systemic thromboembolism. No data are yet available.

This information is meant to provide the reader with some enticing insight with respect to the current horizons in AAD and oral anticoagulant therapy. It is not all inclusive by any means, but I hope it will inform the readers that these subjects are areas of active research and that they should keep on the lookout for more to come.
